# Quantitative Characterization of Bubble Defects in Ultra-Low Expansion Quartz Glass via Ultrasonic Interaction

**DOI:** 10.3390/ma18071639

**Published:** 2025-04-03

**Authors:** Lingxia Zhou, Wenqing Wei, Zisheng Tang, Xue Qi, Zhixiang Wu, Hu Deng

**Affiliations:** 1 School of Information Engineering, Southwest University of Science and Technology, Mianyang 621010, China; 15775792963@163.com (L.Z.); 18381103148@163.com (Z.T.); 17378574923@163.com (X.Q.); zxwu@swust.edu.cn (Z.W.); denghu@swust.edu.cn (H.D.); 2 Tianfu Institute of Research and Innovation, Southwest University of Science and Technology, Chengdu 610299, China; 3 Robot Technology Used for Special Environment Key Laboratory of Sichuan Province, Mianyang 621010, China

**Keywords:** ultra-low expansion quartz glass, bubble defects, interaction analysis, quantitative characterization

## Abstract

The existence of bubble defects in ultra-low expansion quartz glass will affect the optical properties and mechanical strength of the material. The present paper proposes a novel defect characterization method based on ultrasonic nondestructive testing. The simulation model of bubble defect detection in ultra-low expansion quartz glass was established using numerical simulation technology, and experimental verification was carried out. The propagation mechanism of the ultrasound and its interaction with bubble defects were then analyzed. The results showed that the shape of the reflected wave was similar to that of the corresponding defect, and the scattering of the reflected wave was different due to the different curvature radius of the defect interface. The acoustic scattering characteristics of the circular defect were more obvious than those of the elliptical defect. Finally, an analysis of the interaction between different depth defects and different size defects and the ultrasound was conducted, leading to the conclusion that the relative amplitude of the defect echo corresponding to a 6 mm probe diameter shows a monotonic decreasing relationship with the defect depth, and there is also a monotonic corresponding relationship between the relative amplitude of the defect echo and the size of bubble defect. Therefore, it can be concluded that the relative amplitude of the defect echo can be used to characterize the size of the bubble defect. This study not only analyses the interaction between defects and ultrasound but also provides a quantitative characterization of defects using the proposed method.

## 1. Introduction

Ultra-low expansion quartz glass (ULE) is essentially Ti-doped quartz glass, which has excellent optical qualities, mechanical properties, and an extremely low thermal expansion coefficient that approaches zero between 5 and 35 °C [1,2,3]. Because of these special qualities, it is a crucial component for atomic clock cavities, extreme ultraviolet lithography masks, and large-aperture optical systems mirrors, which are used in precision optics, astronomical observation, aerospace, and other domains [1,4,5]. Due to an incorrect melting technique during the ULE preparation process, the externally melted quartz will envelop the internally unmelted quartz raw material, preventing the internal gas from being released on time and resulting in the formation of bubble defects with a random distribution [6]. The presence of bubble defects will impact the material’s mechanical strength, optical qualities, and subsequent processing efficiency.

Visual inspection techniques were previously used by Corning Incorporated to detect internal defects in ULE [7]. Through microscopic investigation, it was confirmed that this approach could detect inclusion defects larger than 80 μm. However, visual inspection is subject to subjective judgment, which can lead to significant random errors, meaning that it cannot meet the current inspection requirements. Non-destructive testing technology is widely used in defect detection because it does not require contact and will not damage the object being tested. Common non-destructive testing technologies used to detect defects include optical coherence tomography (OCT), X-ray computed tomography (XCT), infrared thermal imaging, ultrasonic testing (UT), etc. Frank et al. [8] achieved quantitative characterization of near-surface damage (depth range 14–67 μm) produced during the grinding and polishing processes on optical glass Schott SF6 and Corning Fused Silica, based on high-resolution full-field optical coherence tomography (FF-OCT). However, the depth of detection is limited, and image processing is difficult [9]. Cao et al. [10] successfully detected micron-scale void defects (diameters range 40–95 μm, depth range 0.2–0.4 mm) in 3D-printed 304 stainless steel components by using X-ray computed tomography. But, this technology currently faces challenges, such as high cost and radiation hazards. Infrared thermal-imaging technology detects defects by visualizing heat patterns on the target material [11,12], and its detection results are mainly presented in the form of images. However, this method can only detect surface and near-surface defects [13].

The ultrasonic testing technique is one of the most powerful nondestructive testing techniques for inspecting industrial components [14,15], mainly including scanning acoustic microscopy (SAM), traditional ultrasonic testing, laser ultrasonic testing (LUT), phased-array ultrasonic testing (PAUT), etc. Due to its simple and safe operation and flexible detection method [16], it has been widely used in nondestructive testing technology [17,18] and is widely used in defect detection and quantitative characterization. Zhu et al. [19] successfully achieved non-destructive quantitative characterization of near-surface (depth 80–200 μm) corrosion defects in 7050 aluminum alloy through the combination of scanning acoustic microscopy and binary image-processing technology. But its detection depth is usually limited to 50–500 μm. Qin et al. [20] used laser ultrasonic testing technology and the finite-element method to analyze the interaction between defects and sound waves, proposed a reflection energy coefficient algorithm, and achieved qualitative and quantitative detection of near-surface crack-type defects and circular defects (depth 0.1–0.9 mm). However, compared with ultrasonic detection technology, laser ultrasound has limited detection depth, usually less than 20 mm. Kim et al. [21] used a combination of the finite-element method and ultrasonic testing to quantitatively detect crack defects close to the concrete surface with a depth of 45–70 mm and a width of 0.5 mm. Phased-array ultrasonic testing provides a wider scanning range than traditional ultrasonic testing because the ultrasonic beam may focus and steer. Without altering the probe, phased-array technology can be used to examine objects with intricate geometries [22,23]. This technology is an expensive and complex technology because it uses multiple probes [24].

In traditional ultrasonic testing technology, the impedance mismatch between the air and solid materials necessitates the use of a coupling agent between the probe and the sample to ensure effective ultrasonic wave propagation into the material [25], which complicates automated detection. In order to overcome this obstacle, ultrasonic water immersion nondestructive testing technology has gradually developed [26]. This method employs a water layer of a specific thickness between the probe and the measured object, which enables the stable and reliable transmission and reception of ultrasonic waves. The single-probe transceiver detection method is easy to automate, so it is widely used. Cui et al. [27] used multi-frequency ultrasonic technology (2.25–15 MHz) and water immersion scanning to achieve full-thickness (up to 4.1 cm) quantitative detection and three-dimensional characterization of internal defects (1 μm to 6.35 mm) in samarium cobalt-sintered magnets.

Ultrasound immersion testing has strong penetration and is capable of detecting defects at depths of several centimeters. Coupled with the excellent uniformity and acoustic properties of ULE, the ultrasound waves can propagate reliably and stably, with minimal noise interference, facilitating analysis. This allows for the precise localization and quantification of defects. This makes the technology highly suitable for internal defect detection in ULE materials. Combining ultrasonic testing technology with finite-element simulation can arbitrarily change the sample and internal defect parameters and ultrasonic probe parameters, making it easier to analyze the interaction between ultrasonic waves and defects [28,29].

Therefore, this paper will use ultrasonic nondestructive testing technology and the numerical simulation method to establish the simulation model of ultra-low expansion quartz glass internal bubble defect detection and carry out experimental verification. Then, the ultrasonic propagation characteristics and their interaction with defects are studied. Finally, by analyzing the interaction between different depth defects and different size defects and ultrasonic waves, a quantitative characterization method of bubble defects using the relative amplitude of the defect echo is proposed.

## 2. Materials and Methods

### 2.1. Establishment of Simulation Model

In COMSOL Multiphysics (COMSOL Multiphysics 6.2.0.290) software, the solid mechanics module is used to establish the simulation model of ultrasonic propagation in ULE, and the elastic effect of the solid is the main reason for the fluctuation. In the process of ultrasonic wave propagation in the solid, it follows the momentum equation and the energy conservation equation. The momentum conservation equation describes the momentum change inside the material, while the energy conservation equation describes the energy conversion inside the material. Therefore, in order to simulate the propagation of ultrasonic waves, this model uses the velocity strain formula to solve the governing equation of a general linear elastic material [30], as shown in Equations (1) and (2):

(1) $$\rho \frac{\partial v}{\partial t}-\nabla \cdot S=F_{V},$$

(2) $$\frac{\partial E}{\partial t}-\frac{1}{2}[\nabla_{V}+{\left(\nabla_{V}\right)}^{T}]=0,$$

In the equations, *ρ* represents density; *S* is the stress tensor; *E* is the strain energy; *Fv* is the body force; and *v* is the velocity vector.

**Figure 1:**
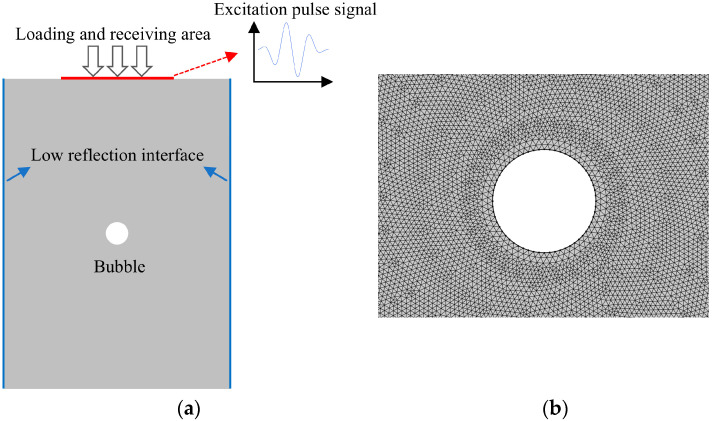
(**a**) Geometric model of the ULE sample with bubble defects. (**b**) Simulation model meshing.

To ensure the efficiency of the simulation calculation, the 3D model is simplified into a 2D section model, as shown in Figure 1a. The geometric size of ULE is 20 × 40 mm, and the defect is preset as a circular bubble with a diameter of 1.5 mm located at the center of the geometric model. The material in the defect area is set to air to simulate bubble defects. To suppress the interference of boundary reflection echoes on the defect echo signal, both side boundaries are set as low reflection boundaries, allowing the ultrasonic wave energy reaching the boundary to be absorbed rather than reflected. The material properties of ULE are listed in Table 1 [31].

The simulation model uses a sinusoidal wave pulse signal modulated by the Hanning window to simulate the actual ultrasonic probe signal [32]. The Hanning window can eliminate the high-frequency interference and energy spectrum leakage of the acoustic pulse, which is closer to the sound field distribution of the ultrasonic transducer. The expression is:

(3) $$x(t)=\frac{1}{2}\left\{\left[1-\cos(\frac{2\pi ft}{3})\right]\sin(2\pi ft)\right\},\;0\leq t\leq 3T,$$

where *t* is the period of the sine wave, and *F* is the center frequency of the excited ultrasonic wave.

While dealing with the problem of acoustic waves, in order to ensure the accuracy of the numerical simulation of ultrasonic propagation, the free triangle grid division form is adopted. And it is required to divide at least 6–12 grids within a single ultrasonic wavelength [33]. Here, 10 grids are divided within a single wavelength:

(4) $$L_{\max}=\frac{\lambda }{10},$$

(5) $$\Delta t=\frac{1}{20f},$$

In the equation, *L*_max_ is the maximum allowable element size, and *λ* is the wavelength of the ultrasonic wave. Here, the calculated maximum element size is 95.56 µm (corresponding to *f* = 5 MHz, *c*_L_ = 5733.9 m/s); the partially enlarged view of meshing is shown in Figure 1b. To ensure the propagation distance of the longitudinal wave does not exceed the length of one mesh element within a single time step, the time step is chosen to satisfy the condition in Equation (4) to ensure simulation accuracy.

### 2.2. Analysis of Ultrasonic Propagation Characteristics and Interaction with Defects

**Figure 2:**
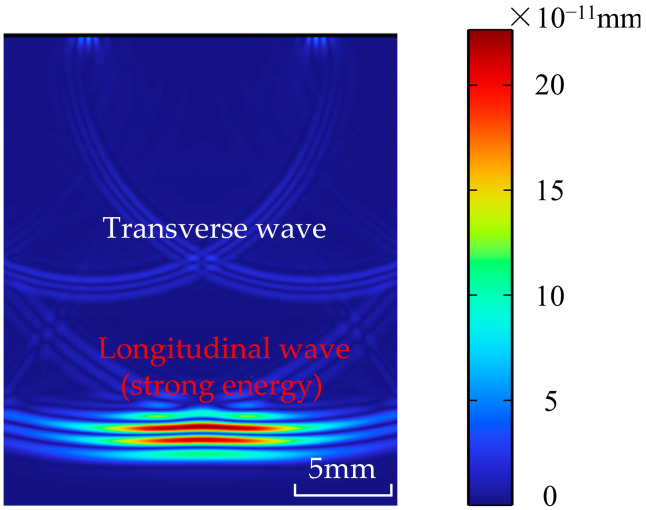
Cloud map of sound field displacement distribution.

The cloud diagram of sound field displacement distribution calculated by the above simulation model is shown in Figure 2. The intensity of ultrasonic energy distribution can be characterized by the color depth in the cloud image of sound field displacement distribution. When propagating in the same medium, the longitudinal wave has the maximum velocity, propagating along the direction of the excitation signal, and the energy is concentrated. The transverse wave velocity is about half of the longitudinal wave velocity, and its energy is weak in the direction of the excitation signal. The longitudinal wave velocity is higher than that of other wave types, does not easily interfere with other wave types, and has strong penetration ability. Therefore, the longitudinal wave is used to detect bubble defects in ULE materials.

**Figure 3:**
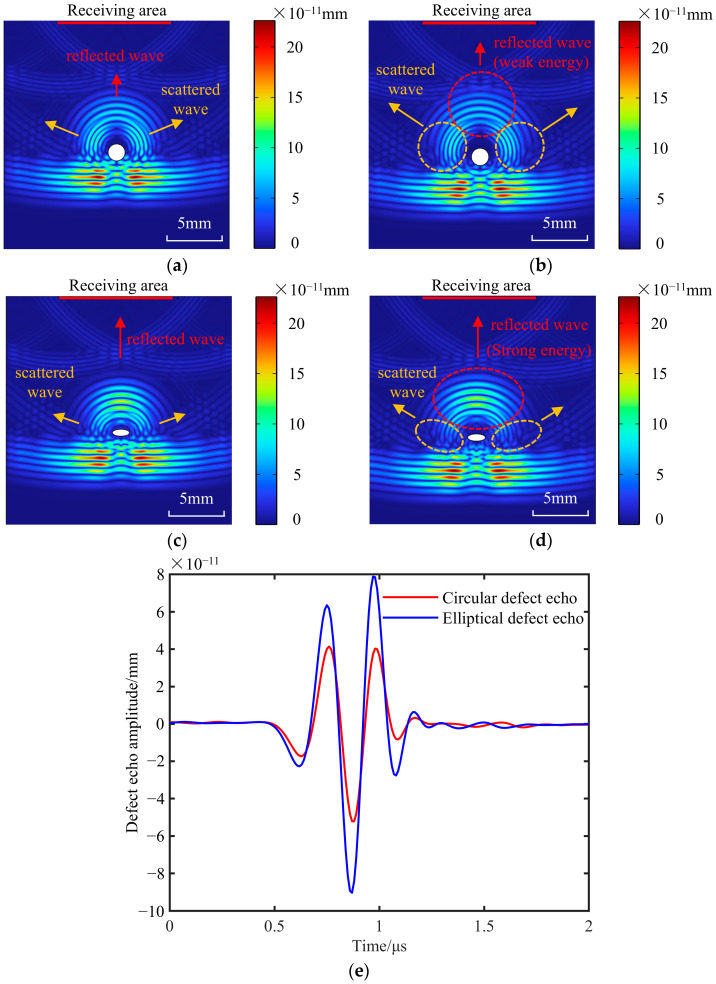
Cloud map of displacement distribution at different times. (**a**) Circular bubble defect, *t* = 4.5 μs. (**b**) Circular bubble defect, *t* = 4.6 μs. (**c**) Elliptical bubble defect, *t* = 4.5 μs. (**d**) Elliptical bubble defect, *t* = 4.6 μs. (**e**) Comparison of echo amplitude between circular defect and elliptical defect.

When ultrasonic waves propagate inside the ULE materials and encounter a circular bubble defect with a diameter of 1.5 mm, the displacement distribution cloud diagrams varying with the time variable t are shown in Figure 3a,b. The longitudinal wave velocity *c*_L_ of the material is known to be 5733.9 m/s, and the calculated longitudinal wave’s wavelength is close to the defect size. So, there is an obvious phenomenon of acoustic diffraction. As demonstrated in Figure 3a,b, the energy of the wave is not fully reflected back by the defect; most waves bypass the defect and continue to propagate in the original direction.

Concurrently, when the ultrasonic wave encounters an elliptical bubble defect with a short semi-axis of 0.3 mm and a long semi-axis of 0.75 mm inside the ULE material, the ultrasonic displacement distribution cloud diagram changes over time, as shown in Figure 3c,d. The circular bubble defect and the elliptical bubble defect both have reflected waves with shapes that are comparable to the respective defects, as can be observed from the reflected wave waveforms in Figure 3a–d. However, the scattering of the reflected wave is distinct due to the variation in the radius of the curvature. When the ultrasonic wave is incident on the circular bubble defect interface, the radius of the curvature of each point on the circle is the same, equal to the radius of the circular defect, 0.75. The ultrasonic scattering characteristics are obvious, and the sound beam energy is dispersed, which leads to the weakening of the defect echo energy. The radius of the curvature of each point on the ellipse is different, which is located between the short half axis 0.3 and the long half axis 0.75. The reflecting interface is relatively flat. The ultrasonic scattering is not obvious, and the defect echo energy is strong. The defect echo amplitude is expressed by the longitudinal displacement of the surface node, and the comparison of the defect echo amplitudes of the circular defects and elliptical defects is shown in Figure 3e.

## 3. Results

**Figure 4:**
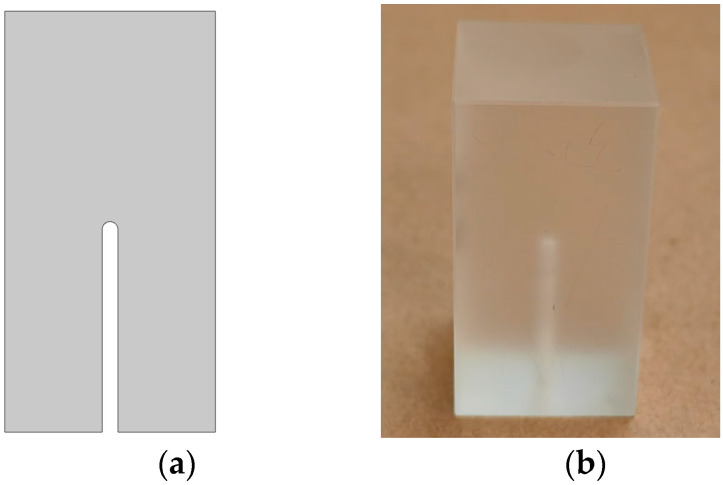
(**a**) Simulation model. (**b**) Physical ULE sample.

**Figure 5:**
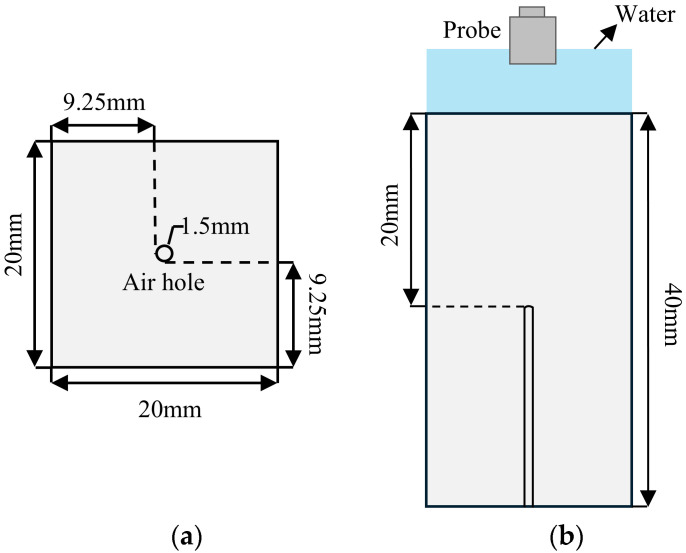
Schematic diagram of defect size and distribution. (**a**) Top view. (**b**) Side view.

Since the difference between the simulation results obtained by the two defect models in Figure 1 and Figure 4a is very small, it can be ignored. And because of the difficulty of actually manufacturing bubble defects, the circular bubble defects in the simulation model will be changed to the form of bottom perforation in the subsequent analysis. To verify the consistency between the experimental results and the simulation, the following experimental parameters are consistent with the simulation parameters for the circular bubble defect. The size of the ULE sample is 20 × 20 × 40 mm, and the air hole is extended to the center of the material to simulate the circular bubble defect in the material. The diameter of the defect is 1.5 mm. The size and distribution diagram of the physical object and defect in the ULE sample are shown in Figure 4b and Figure 5, respectively. The ultrasonic immersion detection method is used in this work. The ultrasonic pulse transceiver (JSR DPR300, Pittsford, NY, USA) sends a narrow pulse signal to the OlympusV326 ultrasonic water immersion probe (Olympus, Tokyo, Japan, probe frequency 5 MHz, probe diameter 10 mm), which causes its internal piezoelectric chip to vibrate and release ultrasonic waves. The ultrasonic waves are reflected back to the ultrasonic probe after making contact with the sample surface and the fault interface. The vibration is converted by the ultrasonic probe into a voltage signal, which is then transmitted via the pulse transceiver for A/D conversion to the data acquisition board. Lastly, the upper computer software displays the data waveform of the signal voltage amplitude changing with time, which is the measured A-scan signal. In the experiment, in order to make the ultrasonic wave act effectively on the defect, the ultrasonic water immersion probe is moved to the top of the ULE sample through a three-axis motion mechanism for single-point detection. The data acquisition card with a sampling rate of 1.25 GHz is used to collect the ultrasonic echo signal. This ensures that the collected signal is as close to the original signal as possible and can capture more subtle defect information, which is conducive to small defect detection. 

In the actual water immersion testing, the ultrasonic wave first enters the water and then enters the material medium. When the thickness of the water layer is small, the near-field region is distributed into two media: water and material. Now, the length of the near-field region of ultrasound in water is converted into the length of the near-field region inside the material to solve the problem of the difference between the detection methods of simulation and measurement. Assuming the thickness of the water layer is *L*, the converted near-field length *N*_1_ and the remaining near-field length *N* in the material medium are [34]:

(6) $$N_{1}=L\frac{c_{1}}{c_{2}},$$

(7) $$N=N_{2}-L\frac{c_{1}}{c_{2}}=\frac{D^{2}}{4\lambda_{2}}-L\frac{c_{1}}{c_{2}},$$

where *c*_1_ is the wave velocity of an ultrasonic wave in water, *c*_2_ is the wave velocity of an ultrasonic wave in the material, *N*_2_ is the length of the near-field region in the material medium, and *λ*_2_ is the medium wavelength of the material. When the water distance *L* is 40 mm, the length of the remaining near-field region in the material medium calculated from Equations (6) and (7) is less than the defect depth of 20 mm, so the defect is located in the non-diffused area of the sound field. The sound pressure distribution in the near-field region is uneven, and the diffusion attenuation in the non-diffusion region is small. So, the experimental verification is carried out in the non-diffusion region. In the simulation model, by changing the defect depth so that the defect is located in the non-diffusion zone, the simulated A-scan waveform signal is characterized by the calculated longitudinal displacement of the surface node.

**Figure 6:**
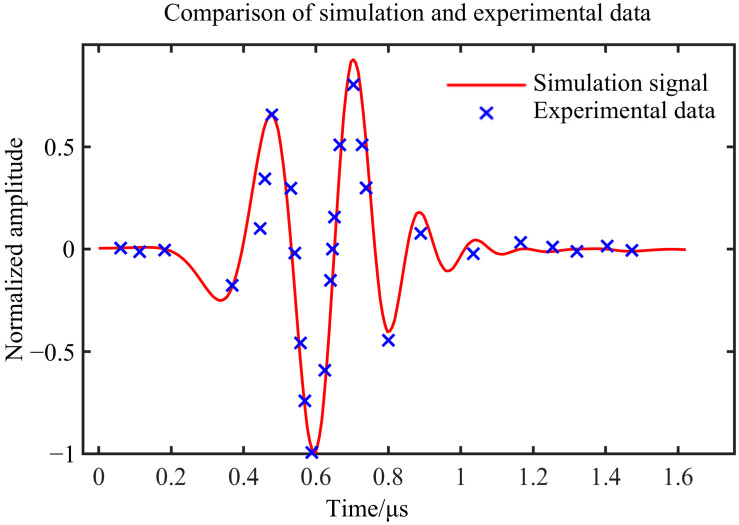
Comparison between experimental data and simulation waveform.

The measured signal is denoised. This paper mainly analyzes the defect echo amplitude, so the defect echo in the A-scan waveform signal obtained from the simulation and measurement is normalized. The comparison results between the experimental data and the simulation waveform are shown in Figure 6.

It can be seen from Figure 6 that the experimental data fit well with the simulated waveform curve. The results show that the simulation can effectively simulate the ultrasonic testing process of the internal defects of the actual ULE material and verify the accuracy of the ultrasonic testing simulation signal. Based on this, the subsequent analysis of the influencing factors will be carried out in the simulation, and the simulation data will be used to study the quantitative characterization method of defects.

## 4. Discussion

### 4.1. Effect of Probe Frequency

The performance of the probe will have a great impact on the quantitative accuracy of defects, so it is necessary to select the appropriate ultrasonic frequency to achieve a better detection effect. According to the near-field region length formula [14], that is, Formula (8), too high a frequency will lead to the near-field region becoming longer and the attenuation increasing.

(8) $$N=\frac{D^{2}}{4\lambda },$$

**Figure 7:**
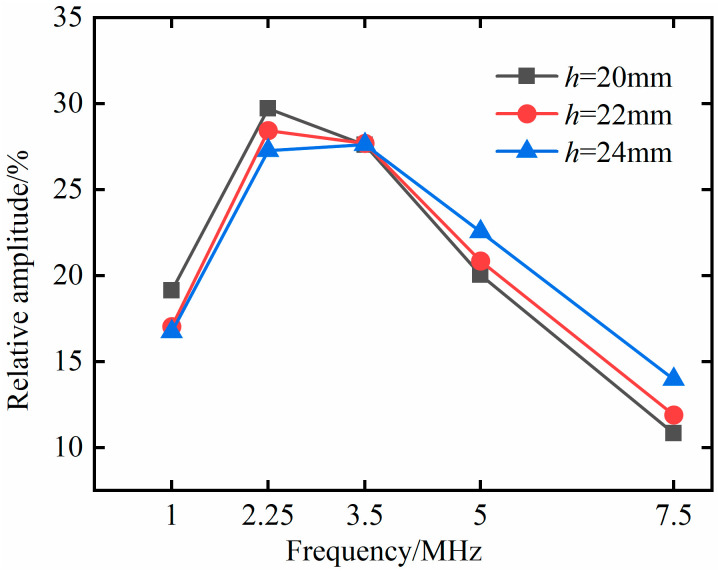
Relationship between the relative amplitude of defect echoes and the probe frequency.

In order to quantify the effect of probe frequency on defect detection, in the above simulation model, the length of the excitation source is set to 10 mm; the defect depth *h* is set to 20 mm, 22 mm, and 24 mm respectively; and the excitation signals with *f* frequencies of 1 MHz, 2.25 MHz, 3.5 MHz, 5 MHz, and 7.5 MHz are selected for simulation analysis. It can be seen from Equation (3) that, when the frequency changes, the amplitude of the excitation source changes. In order to better characterize the defect detection effect of each frequency, the relative amplitude is used to measure the detection effect of the probe frequency, and the relationship between the relative amplitude of the defect echo and the probe frequency is shown in Figure 7.

It can be seen from Figure 7 that, under three different defect depths, the relative amplitude of the defect echo shows the same trend with the change in probe frequency. The relative amplitude of the defect echo shows an increasing trend from 1 MHz to 2.25 MHz, and the relative amplitude of the defect echo corresponding to the 2.25 MHz frequency is the largest. At 3.5 MHz, the three kinds of defects with different depths are located in the range of *N*~1.64*N* (15.26~25.03 mm) in the sound field non-diffusion region, and the relative amplitude has no obvious change. At 5 MHz and 7.5 MHz, as the frequency increases, the wavelength decreases. The defect gradually locates in the near-field region of the sound field at the corresponding frequency, and the relative amplitude of the defect echo shows an obvious downward trend.

### 4.2. Effect of Probe Diameter

According to the above analysis, the relative amplitude of the defect echo corresponding to 2.25 MHz is relatively large, and the excitation signal with a frequency of 2.25 MHz is selected to study the influence of the probe diameter on defect detection. The change in probe diameter is simulated by setting different excitation source lengths. Here, the excitation source lengths of 6 mm, 10 mm, and 13 mm are, respectively, set to simulate the probe diameters of 6 mm, 10 mm, and 13 mm, and the fixed defect depth is 20 mm. The simulation results are shown in Table 2. It can be seen from Table 2 that the relative amplitude of the corresponding defect echo is the largest when the probe diameter is 10 mm, and the relative amplitude decreases when the probe diameter is 6 mm and 13 mm.

**Figure 8:**
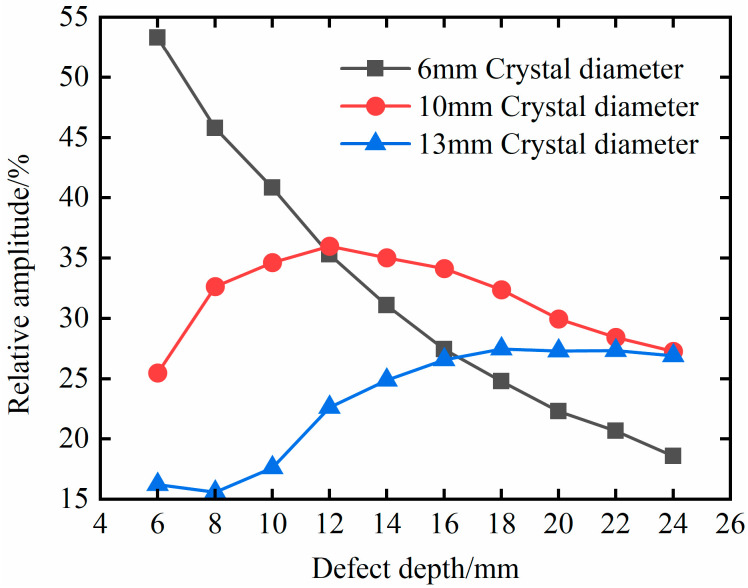
Relationship between the relative amplitude of defect echo and defect depth under different probe diameters.

To further study the influence of probe diameter on the detection distance, change the defect depth, and obtain the simulation results, as shown in Figure 8. It can be seen from the figure that, when the probe diameter is 6 mm, the maximum relative amplitude corresponds to the defect depth of 6 mm, and the relative amplitude gradually decreases with the increase in the defect depth. When the probe diameter is 10 mm, the defect depth is 12 mm, and the detection effect is the best. When the probe diameter is 13 mm, the relative amplitude is the largest when the defect depth is 18 mm, and the detection effect is the best. That is, with the increase in probe diameter, the optimal detection depth increases.

### 4.3. Effect of Defect Depth

**Figure 9:**
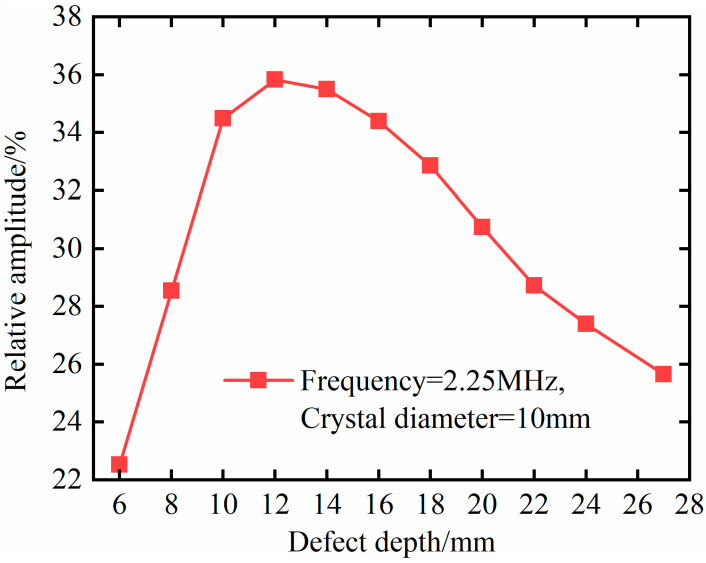
Relationship between the amplitude of defect echoes and the defect’s depth.

In the above 20 × 40 mm geometric model, the frequency of the excitation signal is set to 2.25 MHz, and the length of the excitation source is set to 10 mm. The influence of the defect depth on the defect detection accuracy is simulated and analyzed. According to Equation (8), *N* depends on the probe diameter and frequency. The length of the near-field region corresponding to 2.25 MHz is 9.81 mm. The length of the non-diffusion region is 1.64*N* = 16.09 mm, and the length of the far-field region is 3*N* = 29.43 mm. The depth from the defect center to the excitation source signal is set to 6 mm, 8 mm, 10 mm, 12 mm, 14 mm, 16 mm, 18 mm, 20 mm, 22 mm, 24 mm, and 27 mm, respectively. The corresponding defect echo amplitude results are shown in Figure 9.

It can be seen from the figure that, when the defect depth is 6 mm, 8 mm, and 10 mm, the relative amplitude of the defect varies greatly due to the uneven sound pressure distribution, but the amplitude of the defect echo tends to increase due to the gradual change in the propagation distance from the near-field region to the far-field region. When the defect depth is between 10 mm and 16 mm, the relative amplitude of the defect is relatively high and stable at 34.40~35.83%. When the defect depth is 12 mm, the relative amplitude of the defect echo reaches the maximum, which is due to the small diffusion attenuation in the non-diffusion zone, which can be ignored. The defect depth is between 18 mm and 27 mm, and the relative amplitude gradually decreases from 32.86% to 25.66% due to the diffusion and attenuation in the far-field region with the increase in the propagation distance.

### 4.4. Quantitative Characterization Analysis of Defects

**Figure 10:**
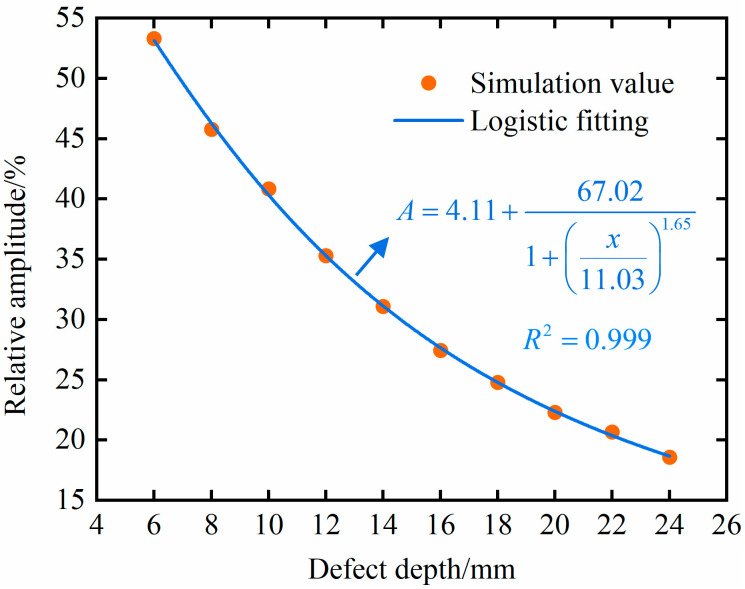
Fitting relationship between relative amplitude and defect depth.

As shown in Figure 8, there is no monotonic correspondence between the relative amplitude of the defect echoes and the defect depth for probe diameters of 10 mm and 13 mm. Therefore, the relative amplitude of the defect echoes cannot be used to characterize defect depth. There is a monotonically decreasing relationship between the relative amplitude of the defect echoes and defect depth under a 6 mm probe diameter. Therefore, the relative amplitude of the defect echoes can be used to characterize the defect depth. And from the data changes in Figure 8, it can be seen that there is a significant nonlinear relationship between the relative amplitude of the defect echoes and the defect depth. Considering the trend of the data changes, this article uses the logistic model for fitting, as shown in Figure 10.

In Figure 10, *x* represents the depth of the defect, and *A* represents the relative amplitude of the defect echo. Use the goodness-of-fit *R*^2^ to measure the fitting effect, where the closer the *R*^2^ is to one, the better the fitting effect. The goodness-of-fit *R*^2^ of this expression is 0.999, indicating its high accuracy.

In order to obtain the size information of bubble defects, in the 20 × 40 mm simulation model, 11 groups of data were taken from the range of a fixed defect depth of 20 mm and a defect diameter from 0.2 mm to 3.5 mm for simulation. The simulation data results showed that the defect amplitude increases with the increase in defect radius, and there is a monotonic correspondence between the relative amplitude of the defect echo and the diameter of the bubble defect. The reason is that the reflected echo energy increases with the increase in defect area. However, when the ultrasonic waves encounters defects, waveform conversion will occur, resulting in reflected waves, scattered waves, etc. So, the relative amplitude of the defect echo does not increase linearly with the increase in bubble defects.

**Figure 11:**
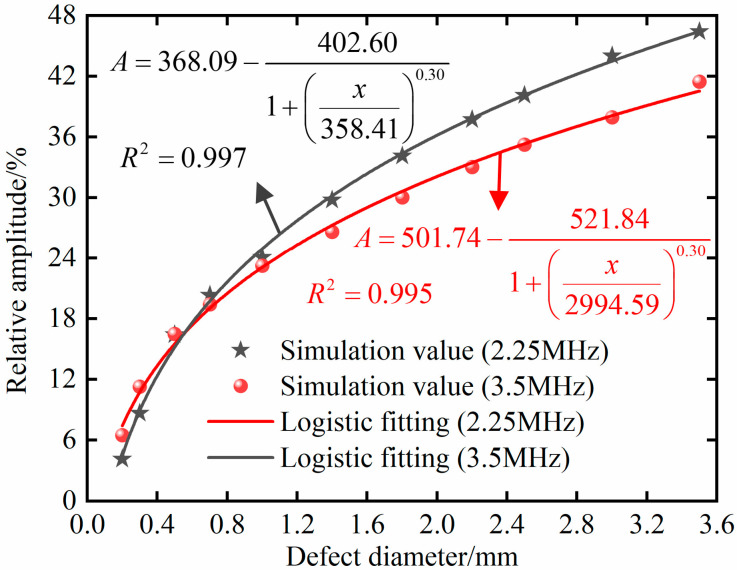
Relationship between relative amplitude of defect echo and defect size in 20 × 40 mm model.

There was also a significant nonlinear relationship between the relative amplitude of the defect echoes and defect size. A logistic model was used for fitting, and the results are shown in Figure 11. The results indicate that both fitting curves have high accuracy, and the goodness-of-fit *R*^2^ is above 0.99.

**Figure 12:**
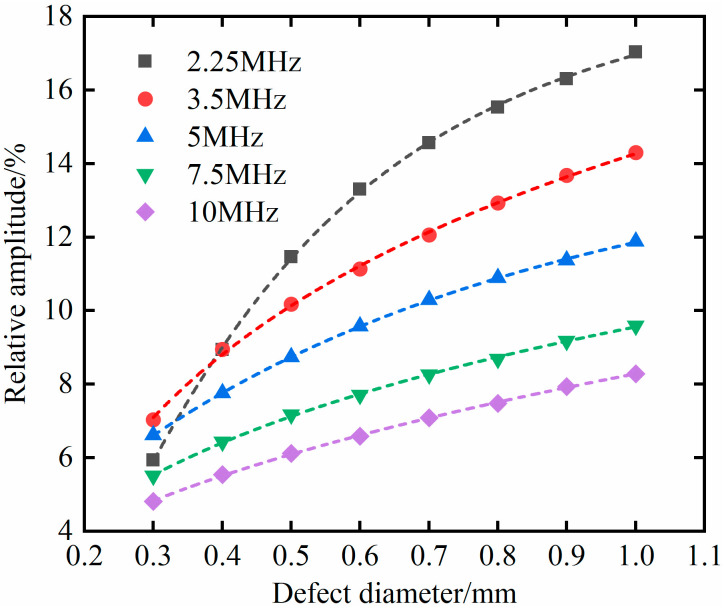
Relationship between relative amplitude of defect echo and defect size in the 80 × 10 mm model.

In the 80 × 10 mm geometric model, set different frequencies, and the other conditions are the same as the above 20 × 40 mm model. The defect diameter changes from 0.3 mm to 1 mm in 0.1 mm increments for simulation analysis. Similarly, the calculated data was fitted using a logic model row, and the results are shown in Figure 12. The results indicate that all five fitting curves have high accuracy, and the goodness-of-fit *R*^2^ is greater than 0.99. And as the frequency increases, the relative amplitude of the defect echo gradually shows a linear relationship with the defect diameter.

In the two geometric models mentioned above, fitting equations were used to obtain fitting prediction values for different defect sizes, which were compared and analyzed with simulation calculation values. The results are shown in Table 3.

From Table 3, it can be seen that the fitting equation has a high prediction accuracy for both geometric bodies. In the 20 × 40 mm geometric model, the maximum difference in predicted values is 2.19%, and the average error value is 1.08%. In the 80 × 10 mm geometric model, the maximum difference in predicted values is 0.15%, and the average error value is 0.09%.

In the geometric models of 20 × 40 mm and 80 × 10 mm, there is a monotonic correspondence between the relative amplitude of the defect echoes and the diameter of the bubble defects. Therefore, the relative amplitude of the defect echoes can be used to characterize the size of the bubble defects. The logistic fitting between the relative amplitude of defect echoes and the diameter of bubble defects has high accuracy and prediction precision.

## 5. Conclusions

In this paper, a two-dimensional model for the detection of internal bubble defects in ULE materials is established by numerical simulation, and the interaction between bubble defects and ultrasound is analyzed. Subsequently, the three parameters of defect depth, probe frequency, and probe diameter are simulated and analyzed, and the quantitative characterization of bubble defects in ULE is proposed by using the relative amplitude of the defect return wave. The main conclusions of this paper are as follows:

(1) By analyzing the interaction between circular and elliptical bubble defects and the ultrasonic waves, it is found that the shape of the reflected wave is similar to that of the corresponding defect. When the ultrasonic wave is incident on the bubble defect interface with the positive radius of curvature, it will produce scattering characteristics, but due to different radii of curvature, the scattering of the reflected wave is different. When the ultrasonic wave is incident on the circular bubble defect interface, the radius of curvature of each point on the circle is the same, which is equal to the radius of the circular defect 0.75, and the ultrasonic scattering characteristics are obvious and the sound beam energy is dispersed, resulting in the defect echo energy being obviously weak. The radius of curvature of each point on the elliptical defect is different, which is located between the short half axis of 0.3 and the long half axis of 0.75. The reflecting interface is relatively flat. The ultrasonic scattering is not obvious, and the echo energy of the defect is strong;
(2) By analyzing the interaction between a bubble defect and an ultrasonic wave at different depths, it is found that the relative amplitude of a defect echo corresponding to a 6 mm probe diameter shows a monotonic decreasing relationship with the defect depth, so the relative amplitude of the defect echo can be used to characterize the defect depth. However, there is no monotonic correspondence between a 10 mm probe diameter and a 13 mm probe diameter, and the relative amplitude of the defect echo cannot be used to characterize the defect depth. By analyzing the interaction between bubble defects with different sizes and the ultrasonic waves, it is found that there is also a monotonic correspondence between the relative amplitude of the defect echo and the size of the bubble defects, so the relative amplitude of the defect echo can be used to characterize the size of the bubble defects. The goodness of fit of the nonlinear fitting curve of the data is greater than 0.99, and it has a high prediction accuracy. In this paper, the interaction between bubble defect and ultrasonic wave is analyzed and verified, and the results show that the bubble defect can be quantitatively characterized by the relative amplitude of the defect echo.

Ultrasonic testing technology has the potential to quantitatively assess defect depth due to its deep penetration and the ability to analyze flight time and amplitude. Different frequency probes can be used to meet different testing needs. It has been widely used in material characterization and even has potential uses in nanoscale systems [35] and material performance evaluation [36,37]. Subsequent research work will conduct multiple sets of experimental verifications to further improve the accuracy and efficiency of ultrasonic testing in the characterization of internal defects in ULE materials, provide more reliable technical support for the quality control of high-precision optical devices, and then, explore high-frequency probes (>10 MHz) to improve the resolution of submillimeter defect characterization.

## Figures and Tables

**Table 1:** Performance parameters of ULE material [31].

Material	Density/(g/cm^3^)	Poisson’s Ratio	Young’s Modulus/(GPa)
ULE	2.21	0.17	67.6
Air	1.29	/	/

**Table 2:** Relative amplitude of defect echoes with different probe diameters.

Probe Diameter/(mm)	Defect Echo Relative Amplitude/(%)
6	22.29
10	29.98
13	27.34

**Table 3:** Comparison between simulated and fitted predicted values.

Geometric Dimensions/mm	Defect Diameter/mm	Simulation Value/%	Estimate/%	Prediction Value Difference/%
20×40 mm	0.45	14.81	15.40	0.59
20×40 mm	0.55	17.79	18.00	0.21
20×40 mm	0.65	19.71	20.25	0.54
20×40 mm	0.75	21.10	22.22	1.12
20×40 mm	0.85	22.15	23.99	1.84
20×40 mm	0.95	23.40	25.59	2.19
80×10 mm	0.45	10.27	10.21	0.06
80×10 mm	0.55	12.46	12.31	0.15
80×10 mm	0.65	13.97	13.89	0.08
80×10 mm	0.75	15.10	15.07	0.03
80×10 mm	0.85	15.86	15.96	0.10
80×10 mm	0.95	16.78	16.64	0.14

## Data Availability

The original contributions presented in this study are included in the article. Further inquiries can be directed to the corresponding author.
